# Self-reported experiences of siblings of children with life-threatening conditions: A scoping review

**DOI:** 10.1177/13674935211026113

**Published:** 2021-06-11

**Authors:** Joanne Tay, Kimberley Widger, Robyn Stremler

**Affiliations:** 1 Lawrence S. Bloomberg Faculty of Nursing, 7938 University of Toronto, Toronto, ON, Canada; 2 Paediatric Advanced Care Team, Hospital for Sick Children, Toronto, ON, Canada; 3 Life Stage Program, ICES, Toronto, ON, Canada; 4 Child Health Evaluative Sciences, Hospital for Sick Children, Toronto, ON, Canada

**Keywords:** Sibling(s), scoping review, critical illness

## Abstract

Sibling relationships are one of the most long-lasting and influential relationships in a human’s life. Living with a child who has a life-threatening condition changes healthy siblings’ experience. This scoping review summarized and mapped research examining healthy siblings’ experience of living with a child with a life-threatening condition to identify knowledge gaps and provide direction for future research. Studies were identified through five electronic databases. Of the 34 included studies, 17 used qualitative methods, four gathered data longitudinally and 24 focused on children with cancer. Four broad themes of sibling experience were identified across studies: family functioning, psychological well-being, social well-being, and coping. Siblings experienced challenges and difficulties over the course of the child’s illness. Future research should incorporate longitudinal designs to better understand the trajectory of siblings’ experiences and focus on a wider variety of life-threatening conditions.

## Introduction

An estimated 21 million children worldwide are living with life-threatening conditions (Connor et al., 2017). Life-threatening childhood conditions take an immense toll on both ill children and their families (O'Haver et al., 2010; Woodgate, 2006). A life-threatening condition is defined as for which ‘curative treatments may be feasible but may fail, or a cure is not possible and from which an affected child is expected to die’ (Spicer et al., 2015: p. 1).

For siblings of children with life-threatening conditions, the illness experience affects their psychosocial well-being, and family and social relationships (Alderfer et al., 2010). Some siblings adjust well to changes brought by illness, while others struggle with these changes (Alderfer et al., 2010; Gan et al., 2017). Siblings may report coping difficulties and poor relationships within the family and at school. These experiences may have long-term impacts on healthy siblings, such as decreased quality of life (Houtzager et al., 2004; Wilkins and Woodgate, 2005).

Despite siblings’ challenges, research and health care focus primarily on the ill child and parents while siblings are referred to as ‘forgotten mourners’ (Alderfer et al., 2010). There has been an increased effort by researchers to understand siblings’ experience, but research remains limited in amount, scope and conclusiveness (Long et al., 2018; Wilkins and Woodgate, 2005). Most research relies on parents’ rather than siblings’ perspectives (Houtzager et al., 2009). While parents’ views may provide useful insights, Houtzager et al. (2009) highlighted significant differences between parent- and sibling-reported quality of life at 1 month and 2 years after diagnosis of childhood cancer in the family. Thus, it is important to incorporate and understand siblings’ perspectives rather than relying on parent reports (Havermans et al., 2015). Additionally, previous reviews focused on siblings of children with cancer (Alderfer et al., 2010). Given that children with cancer make up only about one-third of the population of children with life-threatening conditions (Feudtner et al., 2011), it is important to consider all types of life-threatening conditions to understand siblings’ experiences.

## Aim

To provide a comprehensive overview of research to date and guidance for future research, we conducted a scoping review to summarize and map findings from peer-reviewed research examining healthy siblings’ experiences of living with a child with any life-threatening condition. Specifically, this review addresses the question ‘What is the self-reported experience of siblings of children living with a life-threatening condition?’.

## Methods

The approach was informed by Levac et al.’s (2010) scoping review framework, which is an extension of the original framework by Arksey and O’Malley (2005). This review followed five scoping review stages: (1) identifying the research question, (2) identifying relevant studies, (3) study selection, (4) charting the data, and (5) collating, summarizing and reporting results. We did not include the sixth consultation stage due to a lack of available resources and to be consistent with updated scoping review guidelines (Tricco et al., 2018).

### Search strategy

A search of Medline, Embase, PsychINFO, Social Work Abstracts and the Cumulative Index to Nursing and Allied Health Literature (CINAHL) was initially conducted on 7 June 2017 and then updated on 20 December 2018. Databases were chosen to encompass literature from a broad range of disciplines. Key search terms included the following: palliative care or terminal care or hospice care; life-limiting or life-threatening or life-shortening, end-of-life or end of life care; and sibling* or sister* or brother*. No date limits were set. Reference lists of all included articles were also scanned to identify any additional relevant papers. We excluded conference abstracts given their inconsistency with subsequent full reports (Li et al., 2017). We did not conduct a grey literature search as it did not fit with the focus of our review and interest in mapping the characteristics of peer-reviewed research to identify gaps in approaches and content to guide future research.

### Eligibility criteria

Development of inclusion criteria was an iterative process among three reviewers (JT, KW and LD). During title/abstract review, we excluded papers that were (1) not available in English, (2) conference abstracts, (3) focused on adult patients (>20 years old), (4) focused on siblings of healthy children, or (5) focused only on sudden deaths (e.g. sudden infant deaths, accidents and suicidal and homicide cases). Sudden deaths were excluded as siblings may have different responses compared to death due to a life-threatening condition. During the first full-text review stage, we included articles that reported on siblings’ self-reported experience and excluded articles that reported on (1) both sudden deaths and life-threatening conditions unless results specific to siblings of children with life-threatening conditions could be pulled out, and (2) only parents’ perspectives of the siblings’ experiences, or that reported both parent and sibling perspectives unless siblings’ reports could be separately extracted. After an initial review of full-text papers, we completed a second review with tightened criteria and excluded articles that reported on (1) siblings’ grief experiences, (2) experiences of both bereaved siblings and siblings of an ill child who was still living, unless results specific to siblings of living children could be extracted, (3) interventions, or (4) siblings’ advice to health professionals on how to provide support rather than information about their overall experience.

### Study selection

All identified articles were screened using Covidence – an online screening and data extraction tool to facilitate the review process. Two reviewers (JT and LD) independently screened titles and abstracts of all identified articles and then met to discuss ambiguities related to the broad research question and resolve any selection differences. If no abstract was available, the citation was retained for full-text review. Subsequently, all full texts were reviewed for relevance in two stages, as described above, by two reviewers (JT and KW) independently. Discrepancies were resolved by consensus.

As there is limited research with siblings of children with life-threatening conditions, our aim was to qualitatively synthesize findings from all currently available research and provide direction for future research based both on current knowledge and types of study designs, populations and theoretical perspectives used in current research. As such, a quality assessment of individual studies was not conducted.

### Data charting

Data were initially extracted from each article by one reviewer (JT) and then checked for accuracy by a second reviewer (KW). Discrepancies were resolved by consensus. Extracted data included country of study, study approach (qualitative, quantitative or mixed), study design (i.e. cross-sectional, longitudinal), diagnosis of ill child, number and age range of sibling participants, method of data collection, theoretical models, time of data collection in relation to diagnosis and findings related to siblings’ experiences living with a child with a life-threatening condition.

### Data synthesis

Study characteristics (e.g. approach, design, country and ill child’s diagnosis) were descriptively summarized to create a high-level overview of research conducted to date on siblings’ experiences. Key findings from each study were iteratively reviewed to identify themes across studies.

## Results

**Figure 1.**
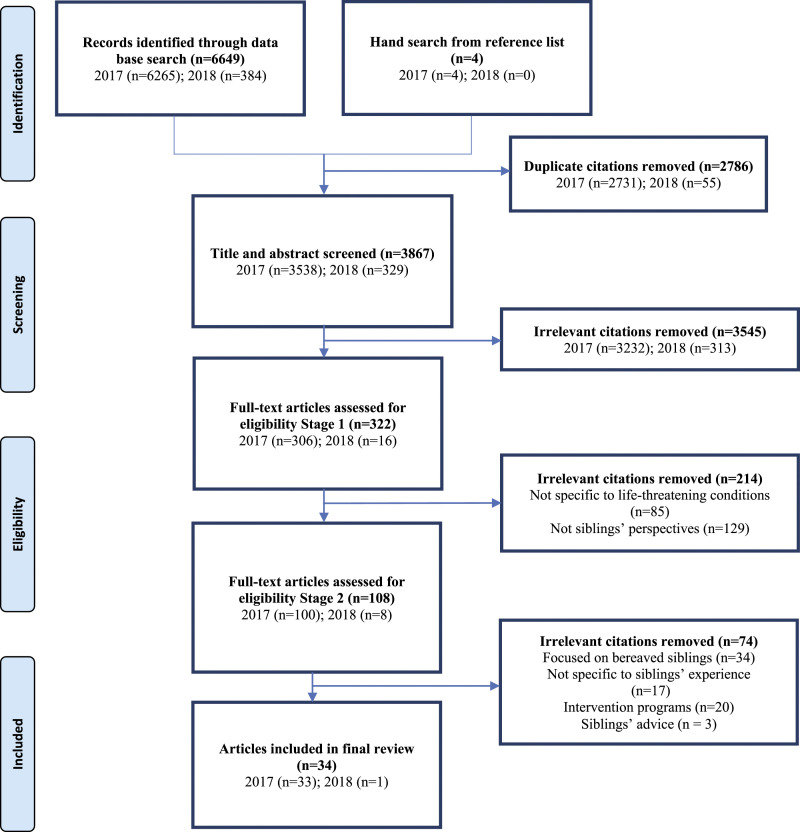
Flowchart outlining the study selection process.

From our initial and updated searches, we identified a total of 6653 potentially relevant articles. After duplicates were removed, we screened 3867 titles and abstracts and identified 332 full-text articles for review. Through a two-stage process, we identified 34 articles relevant to our review question. An overview of the study selection process and reasons for exclusion are provided in Figure 1.

### Characteristics of studies

Across 34 studies in this review, most used a cross-sectional design (88%), were conducted in the United States (47%) and were focused on siblings of children with cancer (70%) (see Supplementary Appendix Table 1). Of the 17 studies that employed solely qualitative approach, 11 were descriptive, while ethnography, phenomenology and grounded theory approaches were used in two studies each. Mixed methods were used in six studies, while 11 used solely quantitative methods. Data extracted from each study are provided in the Supplementary Appendix Table 2.

### Use of theoretical models

Of the 34 studies, only five were guided by a theoretical framework. Three studies (Sloper and While, 1996; Walker, 1988; Wang and Martinson, 1996) adopted Lazarus and Folkman’s Stress, Coping and Appraisal Theory (Lazarus and Folkman, 1984) to examine stress in healthy siblings. Walker (1988) provided little description of how the theory guided development of their siblings’ coping taxonomy. In contrast, Sloper and While (1996) described how the model was used to specifically examine the association between the family’s socioeconomic difficulties and sibling’s behavioural problems. Wang and Martinson (1996) similarly provided an explanation of how they used the model to examine relationships among social factors, demographics and siblings’ behaviour. Packman et al. (2003) combined Erikson’s Developmental Theory (Erikson, 1968) and the Psychosocial Model of Posttraumatic Stress (Green et al., 1985) to examine posttraumatic stress among healthy siblings of children who received a bone marrow transplant. Yin and Twinn (2004) used a family-focused conceptual framework adapted from Northouse (1985) to examine relationships between healthy siblings of children with cancer and other family members.

### Themes

Study findings were synthesized into four broad themes: (1) family functioning, (2) social well-being, (3) psychological well-being, and (4) coping. Family functioning includes three subthemes: (a) disruption in family routine, (b) changes in family environment, and (c) changes in family relationships. Social well-being includes two subthemes: (a) school and (b) community. Each theme or subtheme is described below.

### Family functioning

#### Disruption in family routine

Family routine was disturbed when a child with a life-threatening condition required intensive support (Chesler et al., 1992). Siblings reported parents spending less time with them after the ill child was diagnosed, and they rarely had family activities such as games or movie night or visited grandparents (Evans et al., 1992). Siblings struggled to identify new routines during this difficult period (Evans et al., 1992; Sloper and While, 1996) and expressed their desire to return to the period before illness, or ‘how it used to be’ (Iles, 1979). Loss of family routine that occurred during the time of diagnosis and persisted throughout the illness was described by siblings as depressing, which contributed to a continuous sense of loss (Houtzager et al., 2003).

#### Changes in family environment

Siblings mentioned frequent hospitalizations of the ill child were challenging for them as the family rarely was together (Packman et al., 2003). As parents were rarely at home (Malcolm et al., 2014; Sloper and While, 1996), siblings came back to an empty house which made them feel isolated and lonely (Iles, 1979). Siblings were also introduced to unfamiliar environments such as hospitals and clinics and spent a significant amount of time in these places (Iles, 1979). Siblings also spent some nights at their neighbours’ or friends’ place whenever their parents needed to be in hospital with the ill child (Chesler et al., 1992). Siblings also described having substitute caregivers such as neighbours or grandparents was like coming home to an unfamiliar environment (Iles, 1979). All these created a sense of unpredictability and affected siblings’ overall coping (Hutson and Alter, 2007; Woodgate, 2006).

#### Changes in family relationships

Even though siblings recognized that parents had to care for the ill child, siblings reported feeling overlooked and neglected (Murray, 1998; Read et al., 2010), and that they were treated unfairly and unequally (Chesler et al., 1992; Evans et al., 1992) by parents who overindulged and overprotected the ill child (Stewart and Forrest, 1992). Chesler et al. (1992) found that younger siblings of the ill child often felt displaced within the family as parents were more accommodating towards the ill child. Siblings reported mothers to be more emotional than fathers (Wang and Martinson, 1996); fathers were reported to be more short-tempered throughout the illness period and have more arguments with mothers (Wang and Martinson, 1996). Siblings also described role reversals when fathers assumed more household responsibilities in addition to their work, while mothers spent more time in hospital with the ill child (Houtzager et al., 2004). Nevertheless, some researchers reported improved relationships between siblings and parents over time (Nolbris et al., 2007; Sloper and While, 1996), while others found no change (Evans et al., 1992; Houtzager et al., 2003).

Through qualitative interviews, siblings reported changes in their relationship with the ill child. Deterioration of the ill child’s physical and mental capacity made it difficult for siblings to interact with the ill child and forge ‘normal’ sibling relationships (Brennan et al., 2013). Siblings bore the brunt of the ill child’s fussy or agitated behaviour on bad days (Malcolm et al., 2014). Siblings reported the loss of a playmate as ‘exceptionally difficult’ and ‘life changing’ (Iles, 1979). While some siblings felt frustrated and helpless (Iles, 1979), others developed closer bonds with the ill child and valued time spent together (Nolbris et al., 2014).

### Social well-being

#### School

Siblings expressed fear, frustration and reluctance to share information about the ill child’s condition with teachers or peers due to their lack of information about the illness (Malcolm et al., 2014) and to repeat similar responses when being asked by multiple teachers (Hutson and Alter, 2007). Siblings felt uncared for when others only asked about the ill child’s well-being (Nolbris et al., 2014; Read et al., 2010). Malcolm et al. (2014) and Wang and Martinson (1996) identified bullying as a major concern reported by siblings. Siblings who were teased and bullied by schoolmates expressed confusion about their peers’ behaviour and felt unsupported because teachers failed to intervene in a timely manner (Malcolm et al., 2014). Siblings who were worried about their ill brother or sister were unable to concentrate in school, which affected their school performance (Wang and Martinson, 1996).

#### Community

Siblings reported feeling isolated and neglected by others such as neighbours and family friends as all the attention was on the ill child (Malcolm et al., 2014). While siblings recognized that everyone was concerned with the ill child, they expressed the need to also feel comforted and acknowledged (Evans et al., 1992; Gaab et al., 2014). While many expressed concerns for the ill child, siblings also felt upset and confused when they saw how the wider community did not always show empathy towards the ill child. Siblings witnessed negative attitudes when people avoided or stared awkwardly at the ill child, which indicated low levels of community acceptance towards ill children (Malcolm et al., 2014). Siblings also reported limited social experiences and interaction time with friends as they spent more time at home to be with the ill child (Nolbris et al., 2014; Malcolm et al., 2014). Although siblings were willing to sacrifice their social life to spend time with the ill child, most still hoped to participate in some social activities with friends (Malcolm et al., 2014).

### Psychological well-being

Siblings’ psychological well-being may be affected as they experienced a wide range of negative emotions such as anxiety, fear, distress and sadness (Hamama et al., 2000), and so siblings may be more susceptible to psychological distress at the time of diagnosis or much later in life (Read et al., 2010).

Among all the negative emotions that siblings experience, anxiety was reported in several studies. Stallard et al. (1997) and Packman et al. (2003) reported that siblings of children with life-threatening conditions felt anxious, angry and isolated at time of diagnosis. In relation to gender, adolescent sisters reported higher levels of anxiety throughout the illness compared to sisters of healthy children (Houtzager et al., 2003). However, Hamama et al. (2000) reported that anxiety levels did not differ by age or between brother and sisters. Instead, anxiety was correlated with siblings’ self-control where siblings who had a greater sense of self-control were able to better manage their anxiety.

A common theme across the four longitudinal studies revealed siblings acted as a ‘social-glue’ to help family members remain connected over time (Brennan et al., 2013; Malcolm et al., 2014; Wang and Martinson, 1996; Woodgate, 2006). However, siblings reported experiencing sadness at 6 months post-diagnosis (Brennan et al., 2013). Interviews in Brennan et al. (2013) and Wang and Martinson (1996) studies revealed siblings were committed to protecting the ill child and parents from additional stress by keeping feelings to themselves. Brennan et al. (2013) also found siblings prioritized family needs before theirs to maintain family harmony and characterized themselves as the caring one who helped to relieve parents’ caregiving burden by providing physical and emotional care for the ill child. Despite these efforts, siblings felt they did not contribute much to the family (Woodgate, 2006). Longitudinal studies also indicated that while some siblings showed resilience, others continued to experience emotional and psychological struggles (Brennan et al., 2013). Wang and Martinson (1996) found that though siblings reported normal self-concept scores at baseline and 6 months post-diagnosis, interviews revealed siblings felt unhappy, irritable and anxious at the later time point.

### Coping

Walker (1988) found siblings of children with cancer reported loss, fear of death and change as major stressors and used different strategies to cope with these stressors. Two broad types of coping siblings adopted were cognitive and behaviour strategies. Cognitive strategies included thought stopping, denial and wishful thinking; while behaviour strategies included attention seeking, group activities and taking time out. Although some siblings reported using either behaviour or cognitive coping strategies, Walker (1988) found these strategies were not mutually exclusive. Similarly, Sloper and While (1996) found siblings of children with cancer used a variety of coping strategies such as distraction and wishful thinking to help adjust to disruption brought about by the illness. Brennan et al. (2013) and Malcolm et al.’s (2014) findings further supported that siblings use a mix of strategies to manage stress.

Some researchers examined how siblings’ cope over time. Sargent et al. (1995) reported that over 1 year, siblings changed their coping strategies from containing feelings and stopping thoughts to compartmentalizing their social life from the illness experience. Another longitudinal study reported siblings demonstrated heavy reliance on distraction and wishful thinking although there were no indications if use of these strategies changed over time (Brennan et al., 2013). Similarly, other longitudinal studies found siblings isolated themselves from friends and family (Packman et al., 2003) or distracted themselves by engaging in activities (Gaab et al., 2014). These studies showed that siblings’ coping strategies may or may not change with time.

Siblings cope with feelings of fear and uncertainty by seeking information about the child’s illness through medical pamphlets at the hospital and talking to healthcare professionals (Chesler et al., 1992; Evans et al., 1992). This information offered a sense of self-control, which in turn helped siblings cope with uncertainty (Hamama et al., 2000). However, Malcolm et al. (2014) reported that siblings avoided sharing what they found about the ill child’s condition with their parents for fear of adding to parental stress and burden. Similarly, Martinson et al. (1990) found siblings to have some knowledge of the ill child’s condition but chose not to have an open discussion with parents for fear of causing more stress and sadness.

## Discussion

The purpose of this scoping review was to map and summarize research conducted to date about siblings’ self-reported experiences of living with a child with a life-threatening condition. Most studies were qualitative, and only a few quantitative or mixed methods studies examined relationships between siblings’ experience and their psychosocial outcomes. Studies were also mostly cross-sectional in nature and focused on siblings of children with cancer.

The four broad themes synthesized from across studies highlight the significant impact of a child’s illness on siblings’ lives and their ability to cope. Our findings were similar to reviews that focused on cancer (Alderfer et al., 2010; Wilkins and Woodgate, 2005) or included cancer as part of a variety of chronic illnesses rather than life-threatening conditions (Gan et al., 2017; Sharpe and Rossiter, 2002). In terms of impact on family functioning, Alderfer et al. (2010) also reported that siblings experienced significant disruption to the family’s routine and relationships. In terms of coping and psychological well-being, Wilkins and Woodgate’s (2005) review of qualitative research indicated siblings of children with cancer experienced intense feelings with unmet needs that led to emotional struggle and use of poor coping mechanisms (e.g. acting out at home). For children, much of their social well-being is based on relationships at school. Siblings reported struggles in school, a similar finding to Gan et al. (2017) who attributed overwhelming uncertainties, such as the ill child’s condition, to siblings’ anxiety in school. These findings highlight the need for additional research in this area to identify causes of siblings’ distress and how to best support them.

Research to date primarily focused on siblings of children with cancer; however, the largest group of children with life-threatening conditions is children with a variety of often rare disorders including neurological, metabolic or congenital diseases (Feudtner et al., 2011). Only 10 of 34 studies explored psychosocial well-being in siblings of children with a non-cancer diagnosis. No studies included sub-analyses to determine if there were differences in experience based on the ill child’s diagnosis; thus, it is not clear whether findings may be generalized across disease types. The burden of care in non-cancer conditions may stretch over many years, places immense strain on the families’ physical, financial, and emotional resources and may involve use of technology (e.g. feeding tube and ventilator) to support the child, which may be different than the experience for families of children with cancer (Feudtner et al., 2011; Williams et al., 1999). Other aspects related to diagnosis not yet examined in this body of literature include whether the illness was present at birth or acquired later in childhood and how long the illness lasted. Future research should include siblings of children with a variety of life-threatening conditions – particularly those with non-cancer diagnoses – and large enough sample sizes to permit sub-analyses based on different aspects of the ill child’s diagnosis.

Most study designs were cross-sectional providing an important understanding of siblings’ experience at one time point. However, children with a life-threatening condition may live for many years (Feudtner et al., 2011). Houtzager et al. (2004) argued that cross-sectional studies do not account for factors that may change over time and disregard the dynamic nature of illness and the process of siblings’ adjustment. While qualitative methods were used in both cross-sectional and longitudinal studies, most designs were simply descriptive. Use of more in-depth qualitative methods such as ethnography that may include observation of siblings in their relationships with others over time may help to increase our understanding of the sibling experience and how best to provide support.

Only five included studies incorporated a theoretical framework. Use of theoretical frameworks to guide family or illness-related research helps researchers to contextualize observed problems (Mehta et al., 2009) and enrich interpretation of findings to inform clinical practice and future research. Alderfer et al.’s (2010) review also noted that a lack of theoretical framework reduces the quality of sibling research. Since Lazarus and Folkman’s theory was used in three studies (Sloper and While, 1996; Walker, 1988; Wang and Martinson, 1996), it may be beneficial to continue using this theory to systematically expand existing research on the experience of siblings as individuals. However, given the importance of family relationships in shaping the sibling experience, a family-focused perspective such as Bowen’s Family Systems Theory (Bowen, 1966) may also be beneficial. Adoption of a specific model should be a priority to advance knowledge in this field (Alderfer et al., 2010).

Variations in siblings’ self-reported psychological well-being may be due to differences in measures used across studies and lack of consideration of age and sex differences. Tools to assess siblings’ psychological state included the Behavioral Assessment System for Children (OʼHaver et al., 2010), Piers-Harris Children’s Self-Concept Scale (Stewart and Forrest, 1992) and Kidcope (Brennan et al., 2013; Sloper and While, 1996). Lack of consistency in tools used makes it a challenge to compare results across studies. Only a few studies controlled for age or sex in their analyses (Hamama et al., 2000; Stephenson et al., 2017) which may also contribute to variations in study findings. Alderfer et al. (2010) conducted a systematic review on siblings’ psychosocial adjustment in children with cancer and found some evidence to suggest that age and sex have a significant influence on siblings’ psychological outcomes; therefore, age and sex should be controlled for when examining siblings’ experiences.

Many included studies did not indicate the timing of data collection in relation to time since diagnosis, while others ranged from 1 to 14 years after diagnosis (Brennan et al., 2013; Martinson et al., 1990). Time since diagnosis is important as some research indicates poorer psychological outcomes during early phases of illness compared to later phases (Houtzager et al., 2003). As noted, longitudinal designs are needed to understand siblings’ adjustment over time, but increased reporting of time since diagnosis may help clinicians to anticipate points along the illness trajectory that may be particularly challenging for siblings to navigate. However, it is important to note that in some life-threatening rare diseases, it may take months or years to determine a specific diagnosis; thus, time since the child first became ill or parents first sought medical treatment for the ill child may be a better marker of the beginning of the illness trajectory (Siden and Steele, 2015).

### Limitations

While the search strategy was developed to capture all relevant studies, it is possible that some studies were missed. For example, siblings of children with life-threatening conditions may have been included in research focusing more broadly on the family’s well-being, which may not have been identified through our review process. Similarly, it is possible that some studies may have focused on siblings of children with chronic illnesses but also included siblings of children with life-threatening conditions and thus may have been missed in our search.

### Clinical implications

While the primary goal of this review was to guide additional research in the area, there are some important implications for clinical practice that can be extrapolated from themes synthesized across existing research. To provide support to families of children with life-threatening conditions as early as possible, clinicians should assess the psychosocial state (e.g. family structure and levels of anxiety and depression) of parents and healthy siblings. Clinicians may help parents to understand the need for siblings to feel supported and to maintain family routines as much as possible throughout the illness trajectory. Some life-threatening conditions have an unpredictable life expectancy, which may present additional challenges for siblings. Engaging relevant supports, like child life specialists, early in the disease course to help explain a complex situation in an age-appropriate manner and to develop strategies to cope with uncertainty may be useful. Clinicians can help improve communication and information sharing among the healthcare team, parents and siblings by including siblings during family meetings with healthcare providers. When the ill child’s diagnosis was shared directly with healthy siblings during family meetings with clinicians, siblings and parents expressed their appreciation and satisfaction with the support and information provided (Cordaro et al., 2012). Siblings were able to engage in open communication with parents which facilitated siblings’ adaptation (Cordaro et al., 2012). Clinicians are especially well positioned to educate parents and school staff on potential emotional difficulties that could affect siblings’ social and academic functioning (Gan et al., 2017). Finally, clinicians can help parents to support siblings to develop positive coping strategies over the ill child’s illness trajectory and to recognize when siblings may not be coping well. Some additional supports for siblings may include camps or support groups where they can meet others who may have similar experiences (Barrera et al., 2018) or web-based resources for siblings or parents though some are grief focused (e.g. https://www.dougy.org/; https://courageousparentsnetwork.org/topics/siblings/).

### Future directions

Families play an important role in healthy siblings’ development, overall well-being and coping abilities when there is a child with a life-threatening condition in the family. Future research could consider exploring roles of extended family members (e.g. grandparents), neighbours and the community (e.g. youth clubs and religious or cultural communities) in providing siblings with extra support. These extended social networks may facilitate better support for siblings within an already existing support network and address unique needs of siblings and their family (Alderfer et al., 2010). In addition, future work may consider the influence of cultural, spiritual and religious beliefs or traditions on family’s adaption to help inform future interventions. Researchers should consider designing longitudinal studies on siblings’ psychosocial outcomes or coping and engage more in-depth qualitative approaches. These findings may help parents, clinicians and researchers to recognize patterns of change in siblings over time and form the basis for further development of interventions that are specific to healthy siblings’ unique needs. Longitudinal studies may also shed light on siblings’ resilience and how support may be directed at building siblings’ strengths.

## Conclusion

Siblings of children with a life-threatening condition experience psychosocial changes at some point during the ill child’s disease trajectory. These changes appear to impact the way siblings cope with stress. However, current literature focuses on siblings of children with cancer and study designs are mainly cross-sectional. Examining siblings’ longitudinal experience may help researchers and clinicians to better understand and support siblings and families. In addition, lack of theoretical frameworks or models to contextualize problems that affect siblings’ experience is a barrier to advancing knowledge in this area. As such, future research should employ mixed methods or longitudinal designs to better understand what affects siblings’ experience and how their experiences change over time.

## Supplemental Material

sj-pdf-1-chc-10.1177_13674935211026113 – Supplemental Material for Self-reported experiences of siblings of children with life-threatening conditions: A scoping review Click here for additional data file. Supplemental Material, sj-pdf-1-chc-10.1177_13674935211026113 for Self-reported experiences of siblings of children with life-threatening conditions: A scoping review by Joanne Tay, Kimberley Widger and Robyn Stremler in Journal of Child Health Care
