# Supplementary material for: Self-reported experiences of siblings of children with life-threatening conditions: A scoping review
Source: J Child Health Care. 2021 Jun 11;26(4):517–30. doi: 10.1177/13674935211026113 (PMC9667075; doi:10.1177/13674935211026113)
Supplement: sj-pdf-1-chc-10.1177_13674935211026113 – Supplemental Material for Self-reported experiences of siblings of children with life-threatening conditions: A scoping review [file sj-pdf-1-chc-10.1177_13674935211026113.pdf]

Title: Key Study Characteristics and Findings

Description: This supplementary material highlights the characteristics and findings of all included studies used in this scoping review.

Appendix 1. Key study characteristics and findings based on sibling self-report (arranged chronologically)

Author (Year); Country	Research Aim/Purpose	Number of siblings; age range, mean age (if available); diagnosis of ill child	Study Design	Data collection method(s); measurement tool(s)	Theoretical Model(s)	Time since diagnosis	Findings
Gayton et al. (1977); United States	To examine healthy siblings' psychosocial aspect of living with cystic fibrosis.	26 siblings; 5 to 18 years old; Cystic fibrosis	Descriptive quantitative study	Self-reported survey; Piers-Harris Children's Self-Concept Scale, Missouri Children Picture Series, Holtzman Inkblot Test	None mentioned	Not reported	Younger siblings were described to be more withdrawn than older siblings. There were no differences in self-concept scores that measured school status, behaviour, anxiety, and overall satisfaction between younger and older siblings.
Cairns et al. (1979); United States	To explore the impact of childhood cancer on the ill child and their healthy siblings.	55 siblings; 6 to 16 years old; Cancer	Descriptive quantitative study	Self-reported surveys; Piers-Harris Children's Self Concept Scale, Bene-Anthony, Family Relations Test, Thematic Apperception	None mentioned	Not reported	Siblings described parents to be overprotective of and overindulge the ill child. Overall, siblings expressed anxiety, distress, fear of sharing their feelings with family members and severe stress when living with a brother or sister with cancer. Older siblings were four

Author (Year); Country	Research Aim/Purpose	Number of siblings; age range, mean age (if available); diagnosis of ill child	Study Design	Data collection method(s); measurement tool(s)	Theoretical Model(s)	Time since diagnosis	Findings
				Test			times more likely to express a sense of failure than younger siblings.
Iles (1979); United States	To explore what healthy siblings of children with cancer perceive their experiences to be during various phases of the ill child's illness.	5 siblings; 9 to 11 years old; Cancer	Grounded Theory	Open-ended interview; semi-structured questions	None mentioned	Not reported	Siblings reported disruption in interpersonal family and peer relationships, and changes in the physical and social home environment such as having parent substitutes as caregivers.
Koch-Hattem (1986); United States	To understand siblings' perceptions of changes in themselves and their families after the ill child's cancer diagnosis.	33 siblings; 7 to 18 years old; Cancer	Descriptive qualitative study	Structured and open-ended interview	None mentioned	25 months	Overall, siblings reported feeling worried and sad immediately after learning about the ill child's diagnosis. Anxiety and depression affected their ability to cope with stress.
Walker (1988); United States	To identify and describe cognitive and behavioural coping strategies used by siblings of pediatric oncology patients.	26 siblings; 7 to 11 years old; Cancer	Descriptive qualitative study	Open-ended interview; unstructured questions	Stress, Coping and Appraisal theory	Not reported	Siblings used different strategies to cope with the stress of having an ill brother or sister. Three types of stressors: loss, fear of death and change were identified to affect siblings' coping strategies.

Author (Year); Country	Research Aim/Purpose	Number of siblings; age range, mean age (if available); diagnosis of ill child	Study Design	Data collection method(s); measurement tool(s)	Theoretical Model(s)	Time since diagnosis	Findings
Martinson et al. (1990); United States	To identify the reactions of family members to the experience of having a child with cancer in the family.	16 siblings; 6 to 12 years old/ Cancer	Descriptive qualitative study	Open-ended interview; semi-structured questions	None mentioned	At time of diagnosis	Siblings acknowledged that the ill child needed more attention and parents spent significant amount of time with the ill child, which affected relationships between siblings and parents. Most siblings did not adjust well as they reported feeling jealous, worried and anxious.
Chesler et al. (1991); United States	To report the problems that siblings of children with cancer face, how they dealt with those problems and what advice they had for other siblings of children with cancer.	21 siblings;10 to 21 years old; Cancer	Grounded theory	Semi-structured interview; broad questions	None mentioned	Not reported	Most siblings reported coping difficulties. They were unable to focus at school as they expressed constant worry for the ill child. Pervasive sadness affected the relationship between siblings and parents. However, siblings also reported a growing sense of closeness among family members.
Evans et al. (1992); United Kingdom	To explore siblings' psychological response to childhood cancer and to observe the	16 siblings; 6 to 16 years old; Cancer	Descriptive quantitative study	Self-reported survey; Sibling Questionnaire, Sibling Illness Questionnaire	None mentioned	6 to 18 months	Behavioural problems and low social competence in siblings were related to a lack of knowledge about the ill child's illness. Siblings reported more conflicts with

Author (Year); Country	Research Aim/Purpose	Number of siblings; age range, mean age (if available); diagnosis of ill child	Study Design	Data collection method(s); measurement tool(s)	Theoretical Model(s)	Time since diagnosis	Findings
	effects that sibling knowledge had on their response.						parents at home and did not speak to anyone about their feelings.
Stewart et al. (1992); United Kingdom	To investigate the psychological functioning and family relationships of healthy siblings of children with chronic life-threatening illness.	10 siblings; 6 to 17 years old; Duchenne muscular dystrophy, pulmonary atresia, San fillipo syndrome	Mixed method case control	Self-reported survey and semi-structured interview; Piers-Harris Children's Self Concept Scale, Spielberger Anxiety Rating Scale, Birleson Depressive Rating Scale, Bene-Anthony Family Relations Test (FRT)	None mentioned	Not reported	Siblings showed no difference in emotional and behavioural problems, and self-concept when compared to norms. However, siblings reported that parents' marital adjustment and parental health affected their ability to adjust and cope with stress. FRT scores in siblings were generally lower than the population norms and reflected a poorer father and sibling relationship.
Sargent et al. (1995); United States	To describe siblings' thoughts and feelings about effects of cancer on self and family.	254 siblings; 5 to 18 years old; Cancer	Descriptive qualitative study	Structured interviews; structured questions	None mentioned	6 to 42 months	Majority of siblings reported having coping difficulties immediately after the ill child was diagnosed. Younger sisters reported poorer family relationship and older sisters experienced coping difficulties. Boys in general reported no change.

Author (Year); Country	Research Aim/Purpose	Number of siblings; age range, mean age (if available); diagnosis of ill child	Study Design	Data collection method(s); measurement tool(s)	Theoretical Model(s)	Time since diagnosis	Findings
Sloper & While (1996); United Kingdom	To investigate healthy siblings' adjustment problems following the diagnosis of childhood cancer in the ill child, and to identify the stressors, resources, and coping strategies associated with adjustment problems.	99 siblings; 8 to 16 years old; Cancer	Mixed method	Structured interview and self-reported surveys; Social Support Scale for Children, Self-perception profile for adolescents, Sibling Perception Questionnaire Kidcope	Stress and coping model	6 months	Siblings' negative behaviour was associated with changes in family relationship and environment. Siblings from families with fewer resources reported lower self-perception scores and were more likely to use maladaptive coping strategies such as distraction, blaming others and resignation.
Wang & Martinson (1996); United States	To explore the behavioural responses of healthy Chinese siblings of children with childhood cancer in Taiwan, and to examine the factors that contribute to the behavioural problems in siblings.	45 siblings; 7 to 16 years old; Cancer	Longitudinal mixed method	Semi-structured interview and self-reported survey; Piers-Harris Self Concept Scale	Cognitive theory of psychological stress	Not reported	Siblings identified a lack of family support and poor relationships as major stressors and reported these stressors to affect their coping abilities. Despite these difficulties, siblings reported a sense of family cohesion. Siblings' self-concept scores showed no significant difference between baseline and one year later. However, siblings had higher internalizing

Author (Year); Country	Research Aim/Purpose	Number of siblings; age range, mean age (if available); diagnosis of ill child	Study Design	Data collection method(s); measurement tool(s)	Theoretical Model(s)	Time since diagnosis	Findings
							problems and lower social competence than population norms.
Stallard et al. (1997); United Kingdom	To describe the feelings and beliefs of healthy siblings of children with life-threatening conditions.	52 siblings; 5 to 19 years old; Cystic fibrosis, Central nervous system abnormalities, cardiovascular disease	Mixed method	Semi-structured interview and self-reported survey; Sibling Perception Questionnaire (SPQ), Sibling Questionnaire	None mentioned	Not reported	Siblings coped by keeping to themselves and not talking about their feelings. Younger sisters were more optimistic than older sisters and boys in general. SPQ did not yield significant results related to siblings' behavior.
Murray (1998); United States	To understand the lived experience of one 14-year-old siblings' when the ill sister had childhood cancer.	1 sibling; 14 years old; Cancer	Phenomenolo gy	Open-ended interview; retrospective questions	None mentioned	2 years	The healthy sibling was unable to regulate her emotions at time of the ill child's diagnosis. However, she demonstrated better adjustment over time by using positive coping strategies and providing support to other siblings of children with cancer.
Hamama et al. (2000); Israel	To examine the link between healthy siblings' anxiety and loneliness and self-control when the ill child is	62 siblings; 9 to 18 years old; Cancer	Descriptive quantitative study	Self-reported survey; State-Trait Anxiety Inventory for Children, Loneliness Questionnaire,	None mentioned	Not reported	Siblings had difficulties regulating negative emotions such as anxiety and loneliness. Siblings' age and duration of the ill child's cancer were positively related to anxiety, while

Author (Year); Country	Research Aim/Purpose	Number of siblings; age range, mean age (if available); diagnosis of ill child	Study Design	Data collection method(s); measurement tool(s)	Theoretical Model(s)	Time since diagnosis	Findings
	diagnosed with cancer			Children's Self-Control Scale			siblings' sex and rank in family were positively related to loneliness. Self-control as a coping skill was positively related to anxiety and loneliness.
Sloper (2000); United Kingdom	To explore healthy siblings' feelings and social support they receive when the ill child is diagnosed with cancer.	94 siblings; 8 to 16 years old; Cancer	Descriptive qualitative study	Semi-structured interview; retrospective questions	None mentioned	6 to 18 months	Siblings reported that the loss of companionship and the change in home routine affected their ability to cope with stress. However, these problems were reported to resolve 18 months after the ill child's diagnosis. Siblings reported a reduced involvement in social activities compared to before the ill child was diagnosed. Siblings also reported that teachers and peers at school were an important form of social support.
Lehna (2001); United States	To explore the roles of siblings in families of dying children at home.	8 siblings; 6 to 20 years old; Terminal illness	Ethnography	Observation and participation	None mentioned	Not reported	Siblings coped by taking on new roles as caregivers and school achievers. Siblings reported that assuming these roles distracted them from thinking about the ill child's condition. Siblings reported

Author (Year); Country	Research Aim/Purpose	Number of siblings; age range, mean age (if available); diagnosis of ill child	Study Design	Data collection method(s); measurement tool(s)	Theoretical Model(s)	Time since diagnosis	Findings
							that they took on these responsibilities in order to gain parental attention.
Freeman et al. (2003); United States	To identify commonly reported problems and resources important to siblings of children with brain or spinal cord tumors.	17 siblings; 9 to 30 years old, 17years old; Brain or spinal cord tumours	Descriptive quantitative study	Self-reported survey; Regional survey (Self-designed questionnaire by authors)	None mentioned	10 years	Siblings reported the lack of schoolwork support, and lack of information about ill child's prognosis, pain and preparation for death as important problems. Siblings also reported receiving little to no information from physicians or the health care team.
Houtzager et al. (2003); The Netherlands	To investigate the nature of psychosocial problems in siblings of paediatric cancer patients, and to determine the risk factors associated with the psychosocial problems.	83 siblings; 7 to 18 years old; Cancer	Descriptive quantitative study	Self-reported survey; Youth Self Report (YSR), Dutch Children's Quality of Life questionnaire, State-Trait Anxiety Inventory for Children (STAI-c)	None mentioned	Not reported	Sisters demonstrated higher internalizing behaviors on the YSR scale and anxiety on the STAI-c scale than the healthy Dutch children population. Brothers demonstrated lesser externalizing behavior than the healthy Dutch children population.
Packman et al. (2003); United States	To examine healthy siblings' emotional adjustment	44 siblings; 6 to 18 years old, 11 years; Cancer	Mixed method	Scoring and interpretation of a self-reported tool; Human	Erikson's Development al Theory, Psychosocial	Not reported	Although donor siblings showed more anxiety and lower self-esteem than non-donors, they demonstrated a

Author (Year); Country	Research Aim/Purpose	Number of siblings; age range, mean age (if available); diagnosis of ill child	Study Design	Data collection method(s); measurement tool(s)	Theoretical Model(s)	Time since diagnosis	Findings
	towards Bone Marrow Transplant and living with a child with cancer.			Figure Drawing	model of posttraumatic stress		higher level of adaptive skills than non-donors. Overall, donor siblings experienced school-related problems, felt isolated, angry, depressed, and anxious, and had low self-esteem.
Yin & Twinn (2004); Hong Kong	To explore the effects of cancer on the healthy siblings during different stages of the disease.	11 siblings; 7 to 18 years old, 10 years old; Cancer	Ethnography	Semi-structured interview and field notes; interview guides	Family systems theory	Not reported	Siblings were aware of the ill child’s condition but chose not to talk about it with their parents. Siblings reported that poor communication with their parents placed a strain on the relationship with their parents.
Woodgate (2006); Canada	To understand how childhood cancer and its symptom trajectory are interpreted and experienced by siblings of children with cancer.	30 siblings; 6 to 21 years old, 12 years old; Cancer	Longitudinal qualitative study	Open-ended interview; guided questions	None mentioned	Not reported	Siblings reported that while family cohesion appeared stronger, relationships with parents varied. Siblings took more responsibilities at home but at the cost of their school engagement. Siblings reported absenteeism affected their schoolwork.
Hutson et al. (2007); United States	To explore the experiences of healthy siblings of patients with a	12 siblings; 11 to 21 years old, 13 years old; Fanconi anemia	Descriptive qualitative study	Interview and field notes; guided interview	None mentioned	6 months	Isolation was a common coping strategy used by siblings when they were unable to adjust well.

Author (Year); Country	Research Aim/Purpose	Number of siblings; age range, mean age (if available); diagnosis of ill child	Study Design	Data collection method(s); measurement tool(s)	Theoretical Model(s)	Time since diagnosis	Findings
	chronic genetic disease (Fanconi Anemia).			questions			Siblings attributed poorer relationship with parents to the lack of communication within the family.
Nolbris et al. (2007); Sweden	To understand the healthy siblings' experience of everyday life living with a child with cancer.	10 siblings; 10 to 36 years old/ cancer	Phenomenolo gical-hermeneutic study	Narrative interview; open ended and probed questions	None mentioned	6 months	Siblings experienced adjustment difficulties as they felt conflicted between spending time at home with the ill child or at school. They took on more caregiving roles and were constantly worried about their daily lives. Siblings reported changes in the sibling relationship, but family relationships remained close.
O'Haver et al. (2010); United States	To examine the relationships between risk and protective factors that affect the psychological adaption of healthy siblings of children with cystic fibrosis.	40 siblings; 8 to 17 years old; Cystic fibrosis	Descriptive correlational study	Self-reported survey; Behavioral Assessment System for Children	None mentioned	Not reported	Siblings showed more internalizing than externalizing problems. Adolescent siblings were more withdrawn than younger siblings. Adolescent siblings were also less likely to connect with parents compared to younger siblings.
Read et al. (2010);	To document psychosocial	46 siblings; 11-16 years old, 14.6	Descriptive quantitative	Self-reported survey; General	None mentioned	Not reported	Adolescent siblings had more emotional difficulties

Author (Year); Country	Research Aim/Purpose	Number of siblings; age range, mean age (if available); diagnosis of ill child	Study Design	Data collection method(s); measurement tool(s)	Theoretical Model(s)	Time since diagnosis	Findings
United Kingdom	adjustment in healthy siblings of patients with Duchenne Muscular Dystrophy (DMD).	years old; Duchenne muscular dystrophy	study	Health Questionnaire, Hospital Anxiety & Depression Scale (HADS), and Strengths and Difficulties Questionnaire (SDQ)			reported on the SDQ compared to norms. However, there were no significant differences in anxiety and depression reported on the HADS compared to norms. Approximately half of the siblings reported taking on more caregiving roles to support both parents and the ill child.
Brennan et al. (2012); United Kingdom	To understand the experiences of healthy siblings of children with life-limiting conditions and their coping experiences.	31 siblings; 5 to 16 years old, 9.9 years; Life-threatening illness	Longitudinal mixed method	Self-reported survey; Strength and Difficulties Questionnaire (SDQ), Harter self-perception profile, Kidcope	None mentioned	1 to 14 years	Siblings “compartmentalized” their life in order to feel normal and be able to participate in school or other social activities. Siblings’ self-ratings on prosocial scales placed them on the top end of normative range for SDQ scale where siblings saw themselves as a social glue in peer relationships.
Gaab et al. (2014); New Zealand	To identify the concerns of siblings of pediatric palliative care	18 siblings; 10 to 13 years old; Cancer and heart disease	Descriptive qualitative study	Semi-structured interview; guided questions	None mentioned	6 months to 2 years	Siblings coped by talking to others, which helped them to address their fears and stress. Some reported better adjustment after spending

Author (Year); Country	Research Aim/Purpose	Number of siblings; age range, mean age (if available); diagnosis of ill child	Study Design	Data collection method(s); measurement tool(s)	Theoretical Model(s)	Time since diagnosis	Findings
	patients.						some time with the ill child, while others failed to adjust as they expressed anger and anxiety in managing the situation.
Malcolm et al. (2014); United Kingdom	To report the experiences of healthy siblings of children with rare degenerative and progressive conditions.	8 siblings; 7 to 12 years old, 10.5 years old; Mucopolysacchari doses (MPS) and Batten disease	Longitudinal qualitative study	Semi-structured interview; guided and open-ended questions along with cards.	None mentioned	Not reported	Pain symptoms had an impact on siblings' adjustment. The family's social life was limited, and feelings of sadness persisted within the family over time. Siblings struggled to cope with the long-term effects of the ill child's condition and isolated themselves.
Nolbris et al. (2014); Sweden	To describe siblings' reports of grief related to the experience of having a brother or sister with cancer.	29 siblings; 8 to 24 years old, 14 years old; Cancer	Descriptive qualitative study	Open-ended interview; open-ended questions	None mentioned	6 months to 5 years	Siblings adjusted poorly as they constantly grieve and worry about the ill child. Siblings felt isolated from parents especially when the ill child was hospitalized. Siblings reported poor support from the school as peers teased them about the ill child.
Nolbris et al. (2014); Sweden	To evaluate healthy siblings' experience when a brother or sister	14 siblings; 9 to 22 years old, 14 years old; Cancer	Descriptive qualitative study	Semi-structured interview	None mentioned	Not reported	Siblings with low levels of knowledge about cancer treatment were unable to adjust well. They were more

Author (Year); Country	Research Aim/Purpose	Number of siblings; age range, mean age (if available); diagnosis of ill child	Study Design	Data collection method(s); measurement tool(s)	Theoretical Model(s)	Time since diagnosis	Findings
	is newly diagnosed with cancer.						emotional and reported feeling easily angered by schoolmates and issues related to school.
Fullerton et al. (2017); United Kingdom	To describe the psychological adjustment of healthy siblings of children with life-limiting conditions.	39 siblings; 8 to 16 years old; Congenital heart defects, cystic fibrosis, cerebral palsy, rare chromosomal disorders, muscular dystrophy, dravet syndrome, metabolic disorder, kartageners syndrome	Descriptive quantitative study	Self-reported survey; Paediatric Quality of Life questionnaire (PedsQL)	None mentioned	Not reported	Scores from PedsQL showed that siblings had significantly lower total quality of life, behavioural and emotional scores than population norms.
Stephenson et al. (2017); Canada	To examine mothers' experience of posttraumatic growth on adjustment among healthy siblings of chronically ill children.	70 siblings; 7 to 18 years old; Severe neurological impairment (not yet diagnosed), epileptic encephalopathy/neurodegenerative disease,	Descriptive quantitative study	Self-reported survey; Youth Self-Report	None mentioned	Not reported	Siblings reported that although they wanted to care for the ill child, they felt overwhelmed at times. Adolescent siblings reported more self-internalizing problems.

Author (Year); Country	Research Aim/Purpose	Number of siblings; age range, mean age (if available); diagnosis of ill child	Study Design	Data collection method(s); measurement tool(s)	Theoretical Model(s)	Time since diagnosis	Findings
		mitochondrial encephalomyopathy					
Caris et al. (2018); United States	To assess the impact of hypoplastic left heart syndrome on healthy siblings' quality of life.	32 siblings; 7-30 years old, 12.5 years; Hypoplastic left heart syndrome	Prospective cross-sectional study	Self-reported survey; Sibling Perception Questionnaire (Negative Adjustment Composite Scores; NACS)	None mentioned	Not reported	Siblings' self-reported NACS from the Sibling Perception Questionnaire was low, indicating that siblings did not have many adjustment problems. The NACS was positively correlated with siblings' age where older siblings had poorer adjustment compared to younger siblings.
